# Supplementary material for: Predictors of active ageing among older adults in age-friendly communities in Yanji City, China: A cross-sectional study
Source: PLoS One. 2025 Nov 14;20(11):e0336910. doi: 10.1371/journal.pone.0336910 (PMC12617904; doi:10.1371/journal.pone.0336910)
Supplement: S2 File — (DOCX) [file pone.0336910.s002.docx]

****English: Section A: Intrapersonal Level****

1. No._____________

2. Age: ________ years

3. Gender:

A. male B. female

4. Education level:

A. primary education B. junior high school C. senior high school D. college/ university

5. Marital status:

A. married B. single C. widow/widowed or divorced

6. Living arrangement:

A. living alone B. living with a spouse C. living with offspring D. living with others

7. Income level (family per month):

A. <1000 CNY B.1000-3000 CNY C. 3001-5000 CNY D. > 5000 CNY

****8. Physical Activity****

**8-1. During the past month, on average each week, what type of physical activity did you usually engage in?**
A. Light exercise (no sweating, such as walking, radio calisthenics, gateball, etc.)
B. Low-intensity, not very vigorous exercise (slight sweating, such as recreational volleyball, table tennis, Tai Chi, etc.)
C. Moderate-intensity, more vigorous and sustained exercise (more sweating, such as cycling, jogging, etc.)
D. High-intensity, short-duration exercise causing rapid breathing and heavy sweating (such as badminton, volleyball, basketball, etc.)
E. High-intensity and long-duration exercise with rapid breathing and heavy sweating (such as racing, full-set aerobics, or swimming)

**8-2. How long do you usually maintain each session of the above physical activities? (Choose one)**
A. Less than 10 minutes
B. 11–20 minutes
C. 21–30 minutes
D. 31–59 minutes
E. More than 1 hour

**8-3. In the past month, how often did you perform the above physical activities? (Choose one)**
A. Less than once a month
B. 2–3 times a month
C. 1–2 times a week
D. 3–5 times a week
E. About once a day

**9. Cognitive Function**

| **The Mini-Mental State Exam** | | |
| --- | --- | --- |
| 1. Orientation  Now I will ask you some questions. Most are simple, please answer carefully. | Correct | Incorrect |
| 1. What year is it now? | 1 | 0 |
| 1. What season is it now? | 1 | 0 |
| 1. What month is it now? | 1 | 0 |
| 1. What is today’s date? | 1 | 0 |
| 1. What day of the week is it? | 1 | 0 |
| 1. What city are we in? | 1 | 0 |
| 1. What district are we in? (If patient is from another place, ask which direction their home is located in.) | 1 | 0 |
| 1. What street is this? (If patient is from another place, ask about the street near their home.) | 1 | 0 |
| 1. What floor are we on? | 1 | 0 |
| 1. What place is this? | 1 | 0 |
| 2. Immediate Recall  Now I will tell you the names of three objects. Please repeat them after I finish. (Correct words only, order does not matter.) | Correct | Incorrect |
|  |  |  |
| 1) answered “Ball” | 1 | 0 |
| 2) answered “Flag” | 1 | 0 |
| 3) answered “Tree” | 1 | 0 |
| 3. Attention and Calculation**:**Now please do a subtraction task. Subtract 7 from 100, and keep subtracting 7 from each result. Please tell me each answer until I say “stop.” [Up to 5 subtractions. Score 1 point for each correct answer, regardless of earlier mistakes.] | Correct | Incorrect |
| 1) 100-7=93 | 1 | 0 |
| 2) 93-7=86 | 1 | 0 |
| 3) 86-7=79 | 1 | 0 |
| 4) 79-7=72 | 1 | 0 |
| 5) 72-7=65 | 1 | 0 |
| 4. Delayed Recall**：**Please tell me the three objects I asked you to remember earlier. (Correct words only, order does not matter.) | Correct | Incorrect |
| 1) answered “Ball” | 1 | 0 |
| 2) answered “Flag” | 1 | 0 |
| 3) answered “Tree” | 1 | 0 |
| 5. Naming: What is this? | Correct | Incorrect |
| 1)Watch (accept "clock" or "timepiece") | 1 | 0 |
| 2)Pencil (accept "pen") | 1 | 0 |
| 6. Repetition: Please repeat after me**。** |  |  |
| "Everyone pulls the rope together." | 1 | 0 |
| 7. Reading: Please read this sentence and do what it says (Score 0 if illiterate): | Correct | Incorrect |
| "Close your eyes." | 1 | 0 |
| 8. 3-Stage Command: Here is a piece of paper. Please follow my instructions: | Correct | Incorrect |
| 1) Take the paper with your right hand | 1 | 0 |
| 2) Fold the paper in half | 1 | 0 |
| 3) Put the paper on your left leg | 1 | 0 |
| 9. Writing**：**Please write a complete sentence (must contain a subject and a verb, and convey a complete idea). Score 0 if illiterate. | Correct | Incorrect |
| 1. Copying**：**Please copy the design shown.   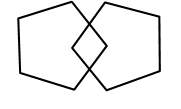 | Correct | Incorrect |

**10. Depressive symptom**

| Geriatric depression scale, (GDS) | |
| --- | --- |
| Please choose the best answer for how you felt during the past week | |
| 1. Are you generally satisfied with your life? | Yes/No |
| 2. Have you cut back on many activities and hobbies (interests)? | Yes/No |
| 3. Do you feel that your life is empty? | Yes/No |
| 4. Are you often bored? | Yes/No |
| 5. Are you in good spirits most of the time? | Yes/No |
| 6. Do your fair bad things to happen to you? | Yes/No |
| 7. Do you feel happy most of the time? | Yes/No |
| 8. Do you often feel incompetent and useless? | Yes/No |
| 9. Do you prefer to stay at home rather than go out and try new things? | Yes/No |
| 10. Do you think you have a worse memory than most people? | Yes/No |
| 11. Do you think it is good to be "alive now"? | Yes/No |
| 12. Do you feel that your life is not worth living right now? | Yes/No |
| 13. Do you feel physically active? | Yes/No |
| 14. Do you feel that your current situation is hopeless? | Yes/No |
| 15. Do you feel that most people are better off than you? | Yes/No |
| Total score |  |

Note: Each item is scored out of 1. Normal is 0 to 5 points; 5 or more points suggests depression.

****Section B: Interpersonal Level****

**11. Family support**

| The Family Functioning Scale (Adaptation, Partnership, Growth, Affection, and Resolve, APGAR) | | | | |
| --- | --- | --- | --- | --- |
| Number |  | Often | Sometimes | Rarely |
| 1 | I can get satisfactory help from my family when I am in trouble. |  |  |  |
| 2 | I'm pleased with how my family discusses various things with me and shares problems. |  |  |  |
| 3 | My family is accepting and supportive when I wish to engage in new activities or developments. |  |  |  |
| 4 | I am satisfied with the way my family reacts to me when I express my emotions and to my anger, sadness, etc. |  |  |  |
| 5 | I'm happy with the way my family spends quality time with me. |  |  |  |

**12. Social connectedness**

| Lubben Social Network Scale -6 （LSNS-6） | | | | | | | |
| --- | --- | --- | --- | --- | --- | --- | --- |
| Dimension | Statements | No | 1 | 2 | 3-4 | 5-8 | 9 and over |
| Family  network | 1. How many family members/relatives do you see or contact at least once a month? |  |  |  |  |  |  |
|  | 2. How many family members/relatives are you comfortable talking to about your personal affairs? |  |  |  |  |  |  |
|  | 3. How many family members/relatives are available to help you when you need it? |  |  |  |  |  |  |
| Friend  network | 4. How many friends do you see or contact at least once a month? |  |  |  |  |  |  |
|  | 5. How many friends are you comfortable talking to about your personal matters? |  |  |  |  |  |  |
|  | 6. How many friends are available to help you when you need it? |  |  |  |  |  |  |

**Section C: Community Level**

1. **Community facilities**

**In the past week, how often have you visited the following community facilities?**

| Facilities type | Never  (0 times) | 1-2 times | 3-5 times | 6-7 times | >7 times |
| --- | --- | --- | --- | --- | --- |
| **Commercial facilities** (e.g., convenience stores, shopping malls, supermarkets, markets) |  |  |  |  |  |
| **Community service facilities** (e.g., community centers, community health service centers, senior activity centers, welfare centers) |  |  |  |  |  |
| **Recreational facilities** (e.g., parks, small open spaces) |  |  |  |  |  |
| **Cultural facilities** (e.g., libraries, civic centers, exhibition centers) |  |  |  |  |  |
| **Religious facilities** (e.g., churches, mosques, temples) |  |  |  |  |  |

**14. Accessibility (by walk):**

The time arrives at ______ (min) market/supermarket, _______ (min) hospital/clinic/health center, ______ (min) public office, _______ (min) senior welfare service center, ______ (min) other welfare service center, ________ (min) bus stop/ subway station facilities.

**Section D: Active Ageing**

1. **Active Ageing Scale (AAS)**

**Please indicate how TRUE each of the following statements is for you?**

| Statements | Not at all true | Slightly  true | Somewhere  at true | Very true |
| --- | --- | --- | --- | --- |
| ***Being self-reliant***  1. I am self-reliant to do all activities in daily living | 1 | 2 | 3 | 4 |
| 2. I try to take care myself before asking others for help | 1 | 2 | 3 | 4 |
| 3. I still work depending upon my competency | 1 | 2 | 3 | 4 |
| 4. Each day, I try to do plenty of activities | 1 | 2 | 3 | 4 |
| 5. I can think or decide with my own autonomy | 1 | 2 | 3 | 4 |
| 6. I help family to do several activities | 1 | 2 | 3 | 4 |
| 7. I can manage for housekeeping with my own arrangement | 1 | 2 | 3 | 4 |
| 8. I like to do leisure time activities to diminish loneliness | 1 | 2 | 3 | 4 |
| ***Being actively engaged with society***  9. I usually participate in public activities or community development activities | 1 | 2 | 3 | 4 |
| 10. I actively participate in elderly club activities or other clubs in which I am a member | 1 | 2 | 3 | 4 |
| 11. I act as a consultant, expert, or local wisdom person within my community | 1 | 2 | 3 | 4 |
| 12. I join ritual or traditional activities within my community | 1 | 2 | 3 | 4 |
| 13.I'm willing to do volunteer work | 1 | 2 | 3 | 4 |
| 14. I transfer my tacit knowledge, wisdom, and skills to others | 1 | 2 | 3 | 4 |
| 15. I like to work for society without concerning for paid | 1 | 2 | 3 | 4 |
| 16. I donate money or materials for community or public benefits | 1 | 2 | 3 | 4 |
| ***Growing spiritual wisdom***  17. I usually look at anything in positive circumstances | 1 | 2 | 3 | 4 |
| 18. I stand firm in my beliefs. | 1 | 2 | 3 | 4 |
| 19. I accept about problems that I cannot solve | 1 | 2 | 3 | 4 |
| 20. I always do good deeds | 1 | 2 | 3 | 4 |
| 21. I try not to attach to anything | 1 | 2 | 3 | 4 |
| ***Building up financial security***  22. I have money or properties enough to meet expenses in later life | 1 | 2 | 3 | 4 |
| 23. I have saved money to use when I am getting old | 1 | 2 | 3 | 4 |
| 24. I have prepared about financial assurance to be used  in my funerary activities | 1 | 2 | 3 | 4 |
| 25. I can provide financial assistance to my family | 1 | 2 | 3 | 4 |
| ***Maintaining healthy lifestyle***  26. I avoid eating sweat, fatty, and salty food | 1 | 2 | 3 | 4 |
| 27. I try to select healthful foods | 1 | 2 | 3 | 4 |
| 28. I regularly eat fish, vegetables, and fruits | 1 | 2 | 3 | 4 |
| 29. I always try to mobilize and stretch my body | 1 | 2 | 3 | 4 |
| 30. I regularly exercise at least 3 times a week engaging in active learning | 1 | 2 | 3 | 4 |
| ***Engaging in active learning***  31. I can learn to use information equipment such as computers and mobile phone | 1 | 2 | 3 | 4 |
| 32. I like to do new things or search for new experiences | 1 | 2 | 3 | 4 |
| 33. I search information to use for taking care of my health | 1 | 2 | 3 | 4 |
| 34. I usually plan to do any activities beforehand strengthening family ties for being cared for in the late  life | 1 | 2 | 3 | 4 |
| ***Strengthening family ties for being cared for in late-life***  35. I have strengthened family ties to maintain children's  attachment when I am getting old | 1 | 2 | 3 | 4 |
| 36. I have taught my children about filial piety and the filial obligation of caregiving to older parents. | 1 | 2 | 3 | 4 |

****Mandarin : Section A: Intrapersonal Level****

1. 编号：

2. 出生年月：

3.性别：A男 B女

4.文化程度：A.小学及以下 B.初中 C.中专或高中 D.大专及以上

5.婚姻状况：A.已婚 B.单身 C.离异、丧偶

6.居住方式：A.独居 B.仅和配偶同住 C.仅和子女同住 D.与其他居住

7.家庭平均月收入：A.＜1000 B.1000～3000 C.3000-5000 D.5000元以上

**8.身体活动**

8-1.过去的一个月里平均每周，您进行体育锻炼的活动通常是:

A、轻微的运动（不出汗，如散步、做广播操、打门球等）

B、小强度的不太激烈的运动（出薄汗，如消遣娱乐性的打排球、乒乓球、太极拳）

C、中等强度的较激烈的持久运动（出汗较多，如骑自行车、跑步等）

D、呼吸急促、出汗很多、大强度的，但并不持久的运动（如打羽毛球、排球、篮球等）

E、呼吸急促、出汗很多、大强度的，且持续很久的运动（如赛跑、成套健美操或游泳）

8-2您进行上述体育活动时每次坚持的时间是:(ABCDE任选其一)

1. ＜10 min B、11～20min C、21～30 min D、3l～59 min E、＞1h

8-3过去一个月里，您进行上述体育锻炼的次数是:(ABCDE任选其一)

1. ＜1次/月 B、2～3次/月 C、1～2次/周 D、3～5 次/周 E、大约1次/天

**9.认知功能**

**简易精神状态评价量表(MMSE)**

| 评价项目 | | | | |
| --- | --- | --- | --- | --- |
| **1.定向力：现在我要问您一些问题，多数都很简单，请您认真回答。** | | 正确 | 错误 | |
| 1. 现在是哪一年? | | 1 | 0 | |
| 1. 现在是什么季节? | | 1 | 0 | |
| 1. 现在是几月份? | | 1 | 0 | |
| 1. 今天是几号? | | 1 | 0 | |
| 1. 今天是星期几? | | 1 | 0 | |
| 1. 这是什么城市（城市名）? | | 1 | 0 | |
| 1. 这是什么区（城区名）?如能回答出就诊医院在本地的哪个方位也可。如为外地患者，则可间患者家在当地的哪个方位） | | 1 | 0 | |
| 1. 这是什么街道（如为外地患者，则可问患者家在当地的哪个街道） | | 1 | 0 | |
| 1. 这是第几层楼? | | 1 | 0 | |
| 1. 这是什么地方? | | 1 | 0 | |
| **2.即刻记忆： 现在我告诉您三种东西的名称， 我说完后请您重复一遍**(答出的词语正确即可， 顺序不要求）。 |  | |  |  |
|  |  |  |  |  |
| 1) 回答出“皮球” | 1 | | 0 |  |
| 2) 回答出“国旗” | 1 | | 0 |  |
| 3) 回答出“树木” | 1 | | 0 |  |
| **3.注意力和计算力:现在请您算一算，从100中减去7, 然后从所得的数算下去，请您将每**  **减一个7后的答案告诉我，直到我说“停”为止**［依次减5次，减对几次给几分，如果前面减错，不影响后面评分，例如：100-7=92(错，本次不得分），92-7=85(对，本次得1分），85-7=78(对，本次得1分），78-7=71(对，本次得1分），71-7=65(错，本次不得分），故本项共得分为3分］。 | 正确 | | 错误 |  |
| 1) 100-7=93 | 1 | | 0 |  |
| 2) 93-7=86 | 1 | | 0 |  |
| 3) 86-7=79 | 1 | | 0 |  |
| 4) 79-7=72 | 1 | | 0 |  |
| 5) 72-7=65 | 1 | | 0 |  |
| **4.回忆：现在请您说出刚才我让您记住的是哪三种东西（回答出的词语正确即司，顺序不要求）？** | 正确 | | 错误 |  |
| 1)回答出“皮球 ” | 1 | | 0 |  |
| 2)回答出“国旗” | 1 | | 0 |  |
| 3)回答出“树木” | 1 | | 0 |  |
| **5.命名：请问这是什么？** | 正确 | | 错误 |  |
| 1)回答出“手表”（回答出“表”就算对） | 1 | | 0 |  |
| 2)回答出“铅笔＂（回答出“笔”就算对） | 1 | | 0 |  |
| **6.重复：请您跟我说。** |  | |  |  |
| 说出“大家齐心协力拉紧绳" | 1 | | 0 |  |
| **7.阅读： 请您念一念这句话， 并按这句话的意思去做（如患者为文盲， 该项评为 0 分）。** | 正确 | | 错误 |  |
| 请闭上您的眼睛 | 1 | | 0 |  |
| **8.3步指令：我给您一张纸，请您按我说的去做。** | 正确 | | 错误 |  |
| 1) 患者右手拿起纸 | 1 | | 0 |  |
| 2) 患者将纸对折 | 1 | | 0 |  |
| 3) 患者将纸放在左腿上 | 1 | | 0 |  |
| **9.表达：请您写一个完整的句子（句子要有主语、谓语，能表达一定的意思）（如患者为文盲，该项评为0分）** | 正确 | | 错误 |  |
| **10.绘图：请您照着这个样子把它画下来**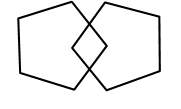 | 正确 | | 错误 |  |

1. **抑郁状态**

| **老年抑郁量表 (GDS)** | | |
| --- | --- | --- |
| **请你为过去的一周内的感受选择最佳答案** | 是 | 否 |
| 1. 您对您的生活基本满意吗？ | 1 | 0 |
| 2. 您减少了很多活动和嗜好（兴趣）吗？ | 1 | 0 |
| 3. 您觉得生活空虚吗？ | 1 | 0 |
| 4. 您常常感到厌倦吗？ | 1 | 0 |
| 5.您是否大部分时间内精神状态都好？ | 1 | 0 |
| 6. 您会害怕将有不好的事情发生在您身上吗？ | 1 | 0 |
| 7. 大部分时间内您觉得快乐吗？ | 1 | 0 |
| 8. 您是否经常感到自己是无能和没用的？ | 1 | 0 |
| 9. 您是否更愿意呆在家里，而不喜欢外出和尝试新鲜事物？ | 1 | 0 |
| 10. 您是否觉得与多数人比较，您的记性更差？ | 1 | 0 |
| 11. 您是否认为“现在还能活着”是一件很好的事情？ | 1 | 0 |
| 12. 您是否感到您现在活得很没有价值？ | 1 | 0 |
| 13. 您觉得体力充沛吗？ | 1 | 0 |
| 14. 您是否觉得您现在的处境没有希望？ | 1 | 0 |
| 15. 您是否觉得大部分人比你过的更好？ | 1 | 0 |

****Section B: Interpersonal Level****

1. **家庭支持**

| **家庭关怀度指数问卷** | | | |
| --- | --- | --- | --- |
| 条目 | 经常 | 有时 | 很少 |
| 1. 当我遇到困难时，可以从家人得到满意的帮助。 | 2 | 1 | 0 |
| 2. 我很满意家人与我讨论各种事情以及分担问题的方式。 | 2 | 1 | 0 |
| 3. 当我希望从事新的活动或发展时，家人都能接受且给予支持。 | 2 | 1 | 0 |
| 4. 我很满意家人对我的情绪表示关心和爱护的方式。 | 2 | 1 | 0 |
| 5. 我很满意家人与我共度时光的方式。 | 2 | 1 | 0 |

1. **社会交往**

| **Lubben 社会网络量表-6 （LSNS-6）** | | | | | | | |
| --- | --- | --- | --- | --- | --- | --- | --- |
| 维度 | 问题 | 没有 | 1个 | 2个 | 3-4个 | 5-8个 | 9个及以上 |
| 家庭网络 | 1. 您一个月至少能与几个家人/亲戚见面或联系？ |  |  |  |  |  |  |
|  | 2. 您能和几个家人/亲戚放心地谈您的私事？ |  |  |  |  |  |  |
|  | 3. 当您需要时，有几个家人/亲戚可以给您提供帮助？ |  |  |  |  |  |  |
| 朋友网络 | 4. 您一个月至少能与几个朋友见面或联系？ |  |  |  |  |  |  |
|  | 5. 您能和几个朋友放心地谈您的私事？ |  |  |  |  |  |  |
|  | 6. 当您有需要时，有几个朋友可以提供帮助？ |  |  |  |  |  |  |

**Section C: Community Level**

**13.社区设施:**

**请问过去一周内去以下社区设施的频率：**

| 设施类型 | 0次 | 1-2次 | 3-5次 | 6-7次 | >7次 |
| --- | --- | --- | --- | --- | --- |
| 商业设施（便利店；商场/购物中心；超市；市场） |  |  |  |  |  |
| 社区服务设施（社区中心/社区卫生服务中心/老年人活动中心/福利中心） |  |  |  |  |  |
| 休闲设施（公园/小型开放空间） |  |  |  |  |  |
| 文化设施（图书馆/市民中心/展览中心） |  |  |  |  |  |
| 宗教设施（教堂/清真寺/寺庙） |  |  |  |  |  |

**14.交通便利性：**

到达 ______ (分钟) 市场/超市、_______ (分钟) 医院/诊所/保健中心、______ (分钟) 社区中心、_______ (分钟) 公园/小型开放空间、______ (分钟) 活动中心/福利服务中心、________ (分钟) 公共汽车站设施的时间。

**Section D: Active Ageing**

**15.积极老龄化**

| **积极老龄化量表** | | | | |
| --- | --- | --- | --- | --- |
| 项目 | 完全不符合 | 有点符合 | 某种程度符合 | 完全符合 |
| 1.我能独立应对一切日常活动 | 1 | 2 | 3 | 4 |
| 2.在寻求别人帮助之前，先尝试独立照顾自己 | 1 | 2 | 3 | 4 |
| 3.我仍坚持做自己力所能及的工作 | 1 | 2 | 3 | 4 |
| 4.我每天坚持做适度的活动 | 1 | 2 | 3 | 4 |
| 5.我能够独立思考或做决定 | 1 | 2 | 3 | 4 |
| 6.我帮助家人做一些家务活动 | 1 | 2 | 3 | 4 |
| 7.我能自己管理好家务 | 1 | 2 | 3 | 4 |
| 8.我喜欢参加休闲活动以减轻孤独感 | 1 | 2 | 3 | 4 |
| 9.我经常参加社会活动或社区发展活动 | 1 | 2 | 3 | 4 |
| 10. 我积极参与老年俱乐部或其他俱乐部的活动 | 1 | 2 | 3 | 4 |
| 11.我是所在社区的顾问、专家或智者 | 1 | 2 | 3 | 4 |
| 12.我参与社区宗教活动或传统庆典活动 | 1 | 2 | 3 | 4 |
| 13.我志愿做一名志愿者 | 1 | 2 | 3 | 4 |
| 14.我想别人传授自己的经验、智慧和技能 | 1 | 2 | 3 | 4 |
| 15.我愿意无偿为社会服务 | 1 | 2 | 3 | 4 |
| 16.我向社区或公共福利机构捐赠钱物 | 1 | 2 | 3 | 4 |
| 17. 我尝尝以积极的心态看待一切事物 | 1 | 2 | 3 | 4 |
| 18.我坚定自己的信念 | 1 | 2 | 3 | 4 |
| 19.我知道有些事情我解决不了 | 1 | 2 | 3 | 4 |
| 20.我总是做好事 | 1 | 2 | 3 | 4 |
| 21.我尽量不依赖任何事情 | 1 | 2 | 3 | 4 |
| 22.我有足够的经济来源以支撑晚年开支 | 1 | 2 | 3 | 4 |
| 23.我已经存了用来养老的钱 | 1 | 2 | 3 | 4 |
| 24.我以为自己的后事做了经济上的准备 | 1 | 2 | 3 | 4 |
| 25.我可以为我的家人提供经济帮助 | 1 | 2 | 3 | 4 |
| 26.我尽量不吃甜食、油腻和过咸的食物 | 1 | 2 | 3 | 4 |
| 27.我尽量选择健康的食品 | 1 | 2 | 3 | 4 |
| 28.我定期吃鱼、蔬菜和水果 | 1 | 2 | 3 | 4 |
| 29.我经常试着运动，伸展身体 | 1 | 2 | 3 | 4 |
| 30.我每周至少坚持锻炼3次 | 1 | 2 | 3 | 4 |
| 31.我会学习使用电脑、手机等新的信息技术和便捷设备 | 1 | 2 | 3 | 4 |
| 32.我喜欢尝试新鲜事物或寻求新体验 | 1 | 2 | 3 | 4 |
| 33.我持续关注健康知识信息 | 1 | 2 | 3 | 4 |
| 34.我常按计划做好手头的工作 | 1 | 2 | 3 | 4 |
| 35.我巩固了维系家庭关系的纽带，一边自己在年老的时候能够有孩子在身边 | 1 | 2 | 3 | 4 |
| 36.我教育孩子们懂得自己照顾年迈的父母时是应尽的义务，要学会尽孝心 | 1 | 2 | 3 | 4 |
