# Supplementary material for: Predictors of active ageing among older adults in age-friendly communities in Yanji City, China: A cross-sectional study
Source: PLoS One. 2025 Nov 14;20(11):e0336910. doi: 10.1371/journal.pone.0336910 (PMC12617904; doi:10.1371/journal.pone.0336910)
Supplement: Attachment — Submitted filename: Response to Reviewer.docx [file pone.0336910.s004.docx]

Dear Dr Elise Rivera and Reviewer,

Thank you for your valuable comments and suggestions on our manuscript entitled “Predictors of Active Ageing among Older Adults in Age-Friendly Communities in Yanji City, China: A Cross-Sectional Study” (PONE-D-25-35732). We have carefully revised the manuscript in accordance with the editor’s and reviewers' feedback. Below, we provide a point-by-point response.

**Editor’s Comments**

**1. Methods:** **Did the authors adjust for any covariates in their models?**

**Response:** Our multiple linear regression analysis was adjusted for key socio-demographic covariates. In our initial submission, the regression results table presented only the variables that were statistically significant in the univariate analyses and were subsequently entered into the final model, for the sake of brevity and clarity. However, we understand that this may have confused.

**To address this, we have now replaced the results table in the manuscript with the complete model output, which includes all variables that were entered into the multiple linear regression, regardless of their statistical significance.** As shown in the updated Table 4 (Page 17, Lines 284), the model was adjusted for covariates, including age, marital status, and living arrangement, in addition to the other significant predictors. This demonstrates that the identified independent predictors (e.g., social connectedness) are essential even after controlling for these potential confounders.

Additionally, we have revised the **Statistical Analysis** section in the Methods to make this adjustment explicit:
" To account for potential confounding effects, the model was adjusted for **key socio-demographic covariates, including age, educational attainment, marital status, living arrangement, and average family monthly income, to isolate the independent effects of the significant predictors on active ageing.**"( Page 11, Lines:227-229 )

**2. Limitations: Please mention the limitations of the cross-sectional study design and use of self-report measures.**

**Response:** We have revised the Limitations section (Page 22, lines 379–382) to acknowledge that the cross-sectional design precludes causal inference, and that reliance on self-report measures may have introduced recall and social desirability bias.

**Response to Journal Requirements:**

**1. Please ensure that your manuscript meets PLOS ONE's style requirements, including those for file naming.**

**Response:** We have carefully reviewed and reformatted the manuscript to ensure it fully complies with PLOS ONE's style requirements.

**2. Please include a complete copy of PLOS’s questionnaire on inclusivity in global research in your revised manuscript.**

**Response:** We have completed the PLOS Questionnaire on Inclusivity in Global Research and will upload it as a Supporting Information file named "S3 Inclusivity in global research questionnaire."

**3. Please remove all personal information, ensure that the data shared are in accordance with participant consent, and re-upload a fully anonymized data set.**

**Response:** We have carefully reviewed our dataset in accordance with the PLOS ONE Data Policy and the guidance outlined in BMJ (2010) “Preparing raw clinical data for publication: guidance for journal editors, authors, and peer reviewers” (BMJ 2010;340:c181), which is referenced on the official PLOS ONE website. The dataset did not contain personally identifying information. To further minimize the risk of indirect identification, the continuous variable “age” has been recoded into age groups consistent with the categories used in the manuscript. All other demographic variables (sex, education level, marital status, living arrangement, and average monthly family income) were retained as categorical variables to replicate the analyses. The dataset is now anonymized and compliant with participant consent and ethical requirements. A revised version has been re-uploaded as **S1_The database anonymized.xlsx**.

**4. If the reviewer comments include a recommendation to cite specific previously published works, please review and evaluate these publications to determine whether they are relevant and should be cited. There is no requirement to cite these works unless the editor has indicated otherwise.**

**Response:** We have carefully reviewed the reviewer comments and confirm that no specific previously published works were recommended for citation. Therefore, no additional references were required or added in the revised manuscript.

**5. Please review your reference list to ensure that it is complete and correct.**

**Response:** We have carefully reviewed our reference list and confirmed that it is complete and correct. All cited works are relevant, and none have been retracted.

**Reviewer’s Comment**

**This is a timely and rigorously conducted study of active ageing in designated Chinese age-friendly communities in Yanji. While there is recognition that no studies have actually evaluated the influence of implementing age-friendly policies in Yanji, it may have enhanced the significance of overall findings if a parallel assessment was undertaken in communities similar in size to Dan Shan and Yuan Fa but without any overtly implemented age-friendly policies.**

**Response:** We greatly appreciate this insightful suggestion. While this comparative design was beyond the scope of the current study, we acknowledge this limitation and have added a statement in the 'Strengths and limitations of the study' section (page 22, lines 384–387). Future studies may consider such comparative approaches to strengthen the evidence.

These revisions have strengthened our manuscript. We sincerely thank you for your thoughtful review and constructive feedback.

Kind regards,
Cheong AiTheng
On behalf of all co-authors
